# Assessing MITRE ATT&CK Risk Using a Cyber-Security Culture Framework

**DOI:** 10.3390/s21093267

**Published:** 2021-05-09

**Authors:** Anna Georgiadou, Spiros Mouzakitis, Dimitris Askounis

**Affiliations:** Decision Support Systems Laboratory, National Technical University of Athens, Iroon Polytechniou 9, 15780 Zografou, Greece; smouzakitis@epu.ntua.gr (S.M.); askous@epu.ntua.gr (D.A.)

**Keywords:** cyber-security culture framework, MITRE ATT&CK matrix, security assessment, detection, mitigation techniques

## Abstract

The MITRE ATT&CK (Adversarial Tactics, Techniques, and Common Knowledge) Framework provides a rich and actionable repository of adversarial tactics, techniques, and procedures. Its innovative approach has been broadly welcomed by both vendors and enterprise customers in the industry. Its usage extends from adversary emulation, red teaming, behavioral analytics development to a defensive gap and SOC (Security Operations Center) maturity assessment. While extensive research has been done on analyzing specific attacks or specific organizational culture and human behavior factors leading to such attacks, a holistic view on the association of both is currently missing. In this paper, we present our research results on associating a comprehensive set of organizational and individual culture factors (as described on our developed cyber-security culture framework) with security vulnerabilities mapped to specific adversary behavior and patterns utilizing the MITRE ATT&CK framework. Thus, exploiting MITRE ATT&CK’s possibilities towards a scientific direction that has not yet been explored: security assessment and defensive design, a step prior to its current application domain. The suggested cyber-security culture framework was originally designed to aim at critical infrastructures and, more specifically, the energy sector. Organizations of these domains exhibit a co-existence and strong interaction of the IT (Information Technology) and OT (Operational Technology) networks. As a result, we emphasize our scientific effort on the hybrid MITRE ATT&CK for Enterprise and ICS (Industrial Control Systems) model as a broader and more holistic approach. The results of our research can be utilized in an extensive set of applications, including the efficient organization of security procedures as well as enhancing security readiness evaluation results by providing more insights into imminent threats and security risks.

## 1. Introduction

In August of 2016, Cybersecurity Ventures predicted that cybercrime would cost the world $6 trillion annually by 2021, up from $3 trillion in 2015 [1]. A prediction that could not have foreseen the COVID-19 crisis [2]. Cybercriminals developed and boosted their attacks at an alarming pace, exploiting the fear and uncertainty caused by the unstable social and economic situation created by the pandemic [3]. According to the UK National Fraud & Cyber Security Centre, Coronavirus-related fraud reports increased by 400% in March 2020 [4] while costing their victims over 800 thousand pounds in one month [5].

Cybercrime costs include data damage and destruction, financial casualties, productivity losses, theft of intellectual property, sensitive data exposure, embezzlement, fraud, post-attack disruption of the normal business course, forensic investigation, restoration and deletion of hacked data and systems, and reputational harm [6,7,8,9]. Understanding the damage caused by cyber-criminals is equally important to comprehend their objectives, motives, and final goals expressed via various adversary patterns and contradicting behaviors [10].

Additionally, as cyber-attacks increase in volume and sophistication, the state of the art of cybersecurity solutions is still lagging behind [11]. Firewalls, antivirus software, intrusion detection systems, security operation centers and numerous others information security tools and countermeasures have been focusing on defending organizations by detecting or preventing attack actions. What the business solutions fail to comprehend is the importance of studying attack action correlations and predicting malicious behavior, allowing for proactive intrusion investigation and mitigation.

The scientific community has been focusing on modeling cybersecurity attack patterns and techniques based on reported incidents and their in-depth analysis in an attempt to forecast adversary behavior, tactical approaches and systematic malicious actions [12,13]. Probably the most recognizable “mid-level adversary model” is the one proposed by MITRE ATT&CK, which addresses the why, how and who is cyber-attacking a digital infrastructure. MITRE ATT&CK is a global knowledge base of adversary tactics and techniques based on real-world observations of cybersecurity threats. It has been widely accepted by both the research society and the industrial domain and has already met numerous applications varying from adversary emulation, red teaming, behavioral analytics development to a defensive gap and SOC maturity assessment. 

In September 2020, McAfee and the University of California, Berkeley’s Center for Long-Term Cybersecurity (CLTC), conducted a survey among security leaders across 325 large- (with more than 5000 employees) and medium-sized (with more than 1000 employees) enterprises in the United Kingdom, United States, and Australia [14]. Fifty-seven percent of the survey respondents were large enterprises, and 62% had an in-house security operations center. Diverse sectors were targeted, including IT, technology and telecoms, retail, transport, financial services, manufacturing and production. Based on their findings, more than 80% of enterprises use MITRE ATT&CK for threat protection [15]. Furthermore, 57% of those participating to the survey declared they use MITRE ATT&CK to determine gaps in deployed security solutions in their enterprise, with 55% recommending it for security policy implementation and 54% using it for threat modeling. 

On the other hand, the scientific community has been exploiting the MITRE ATT&CK knowledge base towards building cyber threat intelligence, focusing on accurately modeling [10,16,17,18,19], detecting [20,21,22], re-generating [23,24,25] or even simulating cyber-attack chains [26,27,28,29]. Discovering ongoing adversary attacks, relating techniques with existing vulnerabilities, comprehending threat notions, steps and ultimate goals has been the main research domain where MITRE ATT&CK matrices found fertile ground. 

Along the above lines, the MITRE ATT&CK framework appears to have found numerous application areas related to cyber-defense and threat emulation, mainly emphasizing comprehending cyber-crime and its approaches. Furthermore, it is being utilized in testing the effectiveness of existing security solutions and products against documented adversary processes. Moreover, current literature focuses on analyzing specific attacks/tactics or specific organization culture and human behavior factors leading to such attacks, while a universal view on the association of both is currently missing.

With a view to addressing this gap, via extensive literature review and research analysis, we have attempted for the first time to bridge a holistic set of cyber-security culture factors (as provided by our developed cyber-security culture assessment framework) with the adversary tactics and threats, as provided by the ATT&CK modeling knowledge base. The cyber-security culture framework assesses the security culture of an organization focusing on the human factor while evaluating all security facets of a business working environment. It has already been used to identify possible insider threat perils [30]. Due to its multi-dimensional approach, it can further assist in identifying gaps in the security policies, procedures, and infrastructure of an enterprise. Gaps that might result in cyber-violations by allowing the application of malicious techniques described and documented in the MITRE ATT&CK matrices. Thus, we attempt to exploit the cyber-security culture framework to identify under-utilized or neglected security mitigations and comprehend possible MITRE ATT&CK risks. 

This paper presents our research work on associating individual and organizational culture dimensions, based on a holistic cyber-security culture framework, with potential adversary behavior and patterns as documented in the MITRE ATT&CK knowledge base. Section 2 presents background information related to both MITRE ATT&CK’s innovative approach towards cyber-crime and our cyber-security culture framework with its multiple levels, dimensions and domains focusing on the human element. Section 3 unfolds our methodological attempt to map the culture model facets to specific mitigation techniques proposed by the hybrid MITRE ATT&CK for Enterprise and ICS matrix. In Section 4, we demonstrate how a cyber-security culture assessment campaign can assist in identifying possible ATT&CK security vulnerabilities via the presented relation matrix using two use case scenarios. In Section 5, we outline a number of considerations and limitations regarding the proposed methodology. Finally, Section 6 concludes with the significance and influence of the proposed methodology for enterprises as well as areas of further research and potential future applications.

## 2. Background

### 2.1. MITRE ATT&CK

**MITRE ATT&CK** was initiated in 2013 in an attempt to document and categorize post-compromise adversary tactics, techniques and procedures (TTPs) against Microsoft Windows systems aiming to improve detection of malicious behavior [31,32]. Over the years, ATT&CK has expanded quite significantly, examining other platforms and technologies, evolving into a knowledge base of cyber adversary behavior and taxonomy for adversarial actions across their lifecycle. It is now being exploited as both an adversary emulation playbook and as a method for discovering analytic coverage and defense gaps inside target networks [33]. 

As a behavioral model, ATT&CK is being based on a number of core components:

- **Tactics**: denoting the tactical adversary objective for performing an attack. It practically addresses the “why” [31,34]. Tactics serve as contextual categories for individual techniques and cover standard, higher-level notations for actions adversaries perform during an attack, such as data exfiltration, privilege escalation and defense evasion [32].
- **Techniques**: describing the means by which adversaries achieve tactical goals by performing an action. In other words, they address the “how” and, in some cases, the “what” an adversary gains by performing an action [11,32]. There may be many ways, or techniques, to achieve tactical objectives, so there are multiple techniques in each tactic category [31].
- **Sub-techniques**: portraying more specific means by which adversaries achieve tactical goals at a lower level than techniques [32,34].
- **Procedures**: attributing the specific implementation the adversary uses for techniques or sub-techniques [34]. They are being used to describe in-the-wild use of techniques or sub-techniques while exhibiting several additional behaviors in the way they are performed [11,33].
- **Mitigations**: defining the countermeasures that could prevent adversaries from achieving their tactical objectives via the usage of specific techniques. Mitigations address the “what to do” about the TTPs (Tactics, Techniques and Procedures) question [35].

Along the above lines, ATT&CK is a knowledge base of adversarial ***techniques*** analyzed into a number of ***sub-techniques***, exhibiting specific ***procedures*** organized into a set of ***tactics***, which outline the key cyber ***mitigation*** techniques. Due to its public adoption by many government organizations and industry sectors, including financial, healthcare, retail, and technology, it has experienced tremendous growth based on contributions from the cybersecurity community [32]. Today, it offers three different models [34]:

1. **ATT&CK for Enterprise**: covering behavior against enterprise IT networks and cloud. The first ATT&CK model was created in September 2013, focusing on the Windows enterprise environment. After refinements and adjustments through internal research, it was publicly released in May 2015 with 96 techniques organized under nine tactics [32]. In 2017, it was expanded to also address Mac and Linux operating systems (apart from Windows). For the first time, it was attributed the name “ATT&CK for Enterprise”.
2. A complementary model called **PRE-ATT&CK** was also published in the same year focusing on the preceding preparation phases, allowing organizations to predict and prepare for attacks before they even happen [36]. In 2019, **ATT&CK for Cloud** was published as part of Enterprise to describe behavior against cloud environments and services [34]. The current model version, released on 27 October 2020, incorporates 14 enterprise tactics analyzed into 177 techniques and 348 sub-techniques provisioning 42 mitigations.
3. **ATT&CK for Mobile**: focusing on behavior against mobile devices (mainly operating Android and iOS platforms). This model was released in 2017, covering techniques involving device access and network-based effects that can be used by adversaries without device access [32,34]. The current version, released on 23 October 2020, consists of 14 tactics analyzed into 86 techniques addressed by 13 mitigations.
4. **ATT&CK for ICS**: characterizing and describing post-compromise adversary behavior while operating within ICS networks [37]. Its development started as a small MITRE research project to apply the ATT&CK structure and methodology to the ICS technology domain due to the increasingly reported cyber-security incidents [38]. In 2017, a review process was initiated, allowing the participation of organizations and individuals from the ICS community to assist in its refinement. It was finally released to the public in January 2020, with its current version (updated on 5 October 2020) numbering 11 tactics, 81 techniques and 50 mitigations.

Having the increasingly multi-dimensional cyber-threats faced by the industrial and critical infrastructures in mind, it becomes apparent that adversaries do not respect theoretical boundaries between IT or ICS when moving across OT networks [39]. Consequently, cyber-security experts soon realized that ATT&CK for ICS and Enterprise models need to be considered in combination. Late in 2020, Mandiant Threat Intelligence, jointly with MITRE, published an article presenting their attempt to **merge MITRE ATT&CK for Enterprise and ICS** to communicate adversary behaviors across OT networks [40]. Thus, presenting a more generalized approach addressing an extended adversary behavior model along with the tools and data formats needed to investigate and analyze reported complex cyber-security incidents of our century.

### 2.2. Cyber-Security Culture Framework

**Figure 1:**
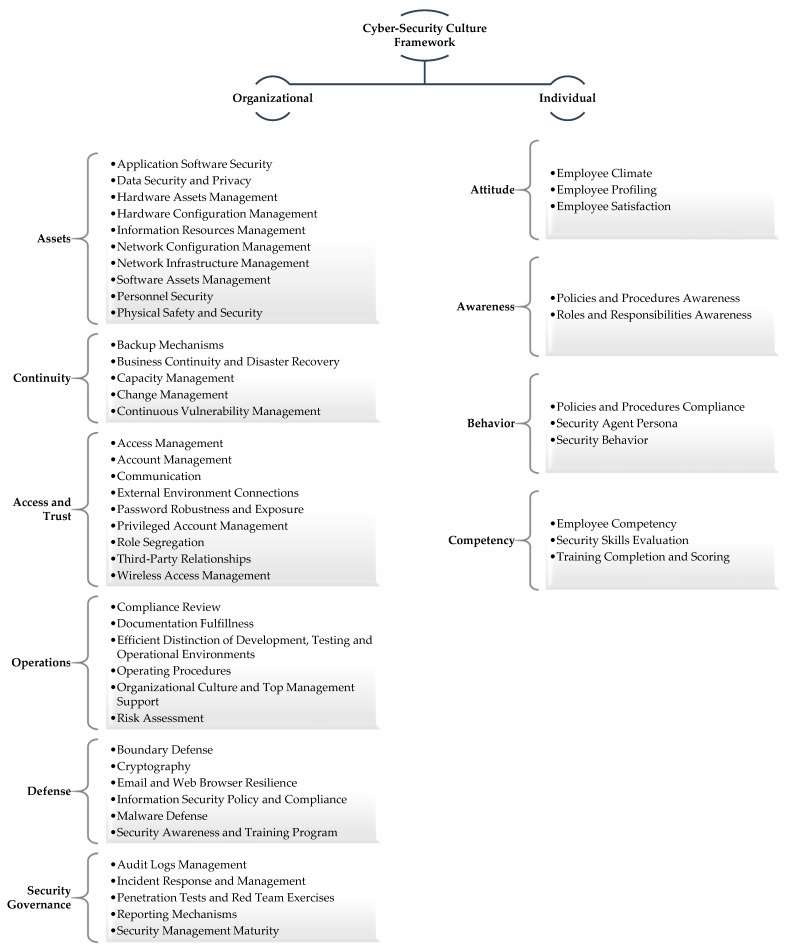
Cyber-Security Culture Framework.

Cyber-Security Culture Framework, presented in 2020 [41], suggests an evaluation and assessment methodology of both individuals’ and organizations’ security culture readiness. It is based on a foundation of both **organizational** and **individual** security factors organized into **dimensions** and **domains,** as depicted in Figure 1. Its elements derive from a thorough and multi-dimensional literature review and research analysis of the current cyber-security reality. It was originally designed to examine organizational security infrastructure, policies and procedures jointly with employees’ individual characteristics, behavior, attitude and skills. Thus, bridging the professional with the scientific approach, the external with the internal indicators directly or indirectly related with cyber-security culture. However, most importantly, a framework co-examining all these security facets with their many interactions and under a complicated business fabric.

Figure 1 presents how the suggested framework organizes security culture indicators into two levels: **organizational** and **individual**. The first level is analyzed into six dimensions:

1. Assets
2. Continuity
3. Access and Trust
4. Operations
5. Boundary Defense
6. Security Governance

Each dimension corresponds to a security facet each organization is meant to address using a combination of IT solutions and security countermeasures. The second level is, in turn, analyzed into four dimensions:

1. Attitude
2. Awareness
3. Behavior
4. Competency

These dimensions are meant to address individual attributes directly affecting the overall security readiness of a business environment. 

Dimensions are further split into **domains** analyzing the different aspects of each security facet. For example, dimension “*Assets*” refers to security policies that enforce several levels of confidentiality, availability and integrity controls on the organization’s assets (including people, buildings, machines, systems and information assets) [41]. Its domains are meant to organize these controls into the different asset categories and security management facets related to them. Therefore, some of the domains met into this dimension are “*Hardware Assets Management”* and *“Hardware Configuration Management,”* “*Network Infrastructure Management*” and *“Network Configuration Management,”* “*Software Assets Management*” and *“Information Resources Management.”* Similarly, each security dimension of this framework presents, in a structured way, the distinctive security application areas of an organization reaching down to quantifiable indicators.

Each domain is then attributed to a number of metrics that can be properly assessed and measured using a variety of evaluation techniques varying from simple surveys and observation techniques and reaching up to more sophisticated methods, such as simulations and serious games. 

A simplified yet targeted and properly trimmed to its purposes survey was carefully designed [42], calibrated, validated and conducted during the COVID-19 pandemic [43], assessing the cyber-security readiness of critical infrastructures during the pandemic. A sophisticated analysis of the collected data [44] demonstrated the complexity and the significance of the suggested evaluation methodology of the cyber-security culture of organizations.

Assessment results offer useful insights to decision-making groups as well as participating individuals contributing to the overall security cultural improvement of the organization while identifying possible cyber-security perils that may endanger the business eco-system. Adversary behavior and the corresponding threats differentiate depending on their origins, meaning inside or outside driven malicious attacks. Identification of potential insider threats based on this framework has already been utilized [30]. This paper presents our effort to detect ATT&CK TTPs’ risks based on the assessment of the presence and utilization of mitigation techniques suggested by MITRE ATT&CK.

## 3. Methodology

Cyber-security culture framework was originally designed as part of the EnergyShield project “Integrated Cybersecurity Solution for the Vulnerability Assessment, Monitoring and Protection of Critical Energy Infrastructures” [45], co-funded under the European Union’s Horizon 2020 research and innovation program. Additionally, although designed and developed under a multi-disciplinary philosophy against security culture, its primary applications were targeted in critical infrastructures, and more specifically, in the EPES (Electrical Power and Energy Systems) sector. 

In 2020, Claroty, a supplier of industrial cybersecurity software, conducted a survey of 1000 IT security professionals around the globe, including the United States, the United Kingdom, Germany, France, and Australia [39]. One of the major findings highlighted in this study was that the survey respondents, who are typically tasked with protecting enterprise networks, are notably more concerned about a cyberattack on critical infrastructure (65%) compared to an enterprise data breach (35%) [46]. Not surprisingly, 80% of global respondents believe it is the IT’s responsibility to protect an organization’s industrial network, while more than half of the industry participants believe that today’s industrial networks are not properly safeguarded and are vulnerable to a cyberattack. 

The prior mentioned security professional notions and trends dictate a combined examination and security evaluation of the IT and OT networks within critical infrastructures. Their co-existence and strong correlation and interaction within the EPES organizations identified the **hybrid MITRE ATT&CK for Enterprise and ICS** approach as the most suitable candidate for the detection of possible external threats. 

The possibility of an adversary achieving a certain tactical goal against a corporate network highly depends on whether certain mitigations have been applied as security countermeasures. To this extend, we have focused on creating a joint MITRE ATT&CK for Enterprise and ICS **mitigations’** list presented in Table 1.

More specifically, this table summarizes the mitigation lists of both ATT&CK for Enterprise [47] and ATT&CK for ICS [48] matrices. Each entry might apply on either one of the models or on both, as marked in the last two columns of the table. In other words, we have created a superset of security countermeasures that need to be properly implemented and addressed in order to safeguard both the IT and OT network of critical infrastructure, such as an enterprise of the EPES sector. 

Having unified the mitigations suggested to protect the IT and OT environments against known ATT&CK TTPs, our next step was to relate them with the suggested cyber-security culture domains and metrics. Thus, evaluation results of the framework would directly identify unutilized mitigation techniques or under-addressed security facets facilitating adversaries into applying specific TTPs. For this association, we have conducted a literature review and in-depth analysis of each security asset and possible tactics and security threats. Table 1 presents how starting from the evaluation of specific security dimensions and domains one can proceed in detecting unapplied or poorly materialized mitigations. The hybrid MITRE ATT&CK for Enterprise and ICS matrix assists us to proceed further by highlighting the possible adversary techniques the organization lacks resilience against [40].

Table 2 presents a many-to-many relationship between the cyber-security culture model and the hybrid MITRE ATT&CK for Enterprise and ICS mitigation list. Seemingly, the assessment results of many different security domains need to be jointly examined to evaluate the fulfillment of a number of mitigations along with the risk of numerous possible ATT&CK TTPs.

The cyber-security culture framework has been created using a multidisciplinary approach towards information security. Therefore, its elements are meant to attribute all different aspects of a business environment, including internal and external, organizational and individual factors. MITRE ATT&CK, on the other hand, has been developed based on an extensive knowledge base of witnessed and documented violation incidents mainly related to technological-driven techniques. In other words, it is meant to describe how adversaries can take advantage of specific IT and OT vulnerabilities and weaknesses to achieve certain malicious goals. Consequently, cyber-security culture, due to its originating purposes, bears a broader nature than ATT&CK. Therefore, detection of MITRE ATT&CK risk does not require the evaluation of all dimensions and domains of the cultural framework. At least, in its current version, since the ATT&CK knowledge base is constantly evolving following the concurrent cyber-crime transformation.

As witnessed in Table 2, all six of the organizational dimensions participate in the ATT&CK risk assessment but without exploiting all sub-domains. Similarly, at the individual level, only two out of four dimensions are used. “Attitude” and “Awareness,” deriving from humanitarian sciences, are not immediately related to TTPs. These dimensions are, on the other hand, used to identify the Insider Threat [30], which is not practically addressed using the ATT&CK technical approach. 

To summarize the above, Table 2 reveals how starting from an overall security assessment of an organization, using a structured evaluation methodology, one can exploit results related to specific security indicators to identify which security measures have not been properly implemented. Thus, understanding the ATT&CK TTPs the organization is vulnerable against.

## 4. Use Case Scenarios

To demonstrate how starting from the cyber-security culture assessment results, one can identify possible ATT&CK vulnerabilities and obtain valuable insights on materializing further security measures, we analyze two application scenarios:

### 4.1. Simple Scenario

Mr. X is responsible for one of the main electricity substations of a TSO (Transmission System Operator) company. **“*Physical Safety and Security”*** along with ***“Personnel Security”*** are critically important for the seamless operation of the facility. Therefore, he periodically runs evaluation campaigns via the cyber-security culture framework, aiming to assess these specific domains of the ***“Assets”*** dimension. During the last invocation, though, disturbing results were noticed related to the **“*Physical Safety and Security”*** domain. Subsequently, the framework suggested re-examining mitigations ***“M0805—Mechanical Protection Layers”*** and ***“M0812—Safety Instrumented Systems”*** while alerting for a number of possible cyber-threats, such as ***“T0879—Damage to Property”*** and ***“T0880—Loss of Safety.”*** Additional technique information, useful references, and procedure examples deriving from the MITRE ATT&CK knowledge further enlightened Mr. X leading him to proper immediate actions toward improving his facility’s security and safety.

### 4.2. Complex Scenario

A power generator company, prior to its annual ISO (International Organization for Standardization) 27001 inspection, designs and runs an evaluation campaign aiming to assess the organizational ***defense*** using a variety of delivery methods provisioned by the cyber-security culture framework. Results demonstrate a low achievement score related to the ***“Security Awareness And Training Program”*** domain suggesting mitigation ***“M1017—User Training”*** might be endangered. Based on MITRE ATT&CK matrix, numerous adversary techniques and sub-techniques addressed by specific mitigation become possible active risks, such as **“*T1003—OS Credential Dumping,*” “*T1176—Browser Extensions*”** and **“*T1185—Man in the Browser.*”** Mitigation M1017, as presented in Table 2, is further assessed by individual-level domains related to employee competency and security behavior. Consequently, the security management team should further proceed in examining these security domains to understand to what extend is M1017 jeopardized. In parallel, identified TTPs threatening the organization need to be additionally probed by investigating the rest of the contributing mitigations addressing them. 

Use the case scenarios presented previously indicatively demonstrate the complexity of the cyber-security culture assessment challenge and the intense interaction and co-existence of various security factors when facing complicated and technologically advanced organizations and networks.

## 5. Considerations and Limitations

The cyber-security culture framework was originally designed to apply to any size and kind of organization regardless of its business domain, specialization, technological status, and security readiness [41]. Its adjustment and fine-tuning to the EPES sector simply meant enriching controls and security indicators used with special ones related to the power supply chain operation lifecycle. On the other hand, ATT&CK is a breakdown and classification of offensively oriented actions that can be used against specific platforms [31] and, as such, is meant to focus on how adversaries interact with systems during an attack. It is a detailed knowledge base presenting rather specialized information. The transition from generic dimensions and domains evaluating a variety of security facets to particular mitigations and adversary techniques aiming at specific vulnerabilities related to enterprise and ICS components dictates human intervention. Security experts need to examine, analyze, and further elaborate on TTPs suggested by the framework while taking into consideration enriched information provided by the MITRE ATT&CK knowledge base. 

Furthermore, adversary behavior adapts to economic, societal, political, and technological circumstances taking advantage of any vulnerability, gap or deficiency of both digital infrastructures and human operators. As a result, information security needs to grow and evolve proportionally. Both the cyber-security culture framework and MITRE ATT&CK knowledge base are living mechanisms continuously evolving, maturing and adjusting to the constantly transforming cyber-crime reality [49]. Consequently, the effort presented in this article, in order to keep up the pace, requires constant revision and enrichment to properly relate these two models.

## 6. Conclusions and Future Work

Cyber-crime is constantly rising and transforming, hardening current rather demanding business reality. An effective defense demands a profound comprehension of the notions, techniques and practices motivating and facilitating adversaries to succeed in their ultimate tactical goals. The MITRE ATT&CK framework is a comprehensive matrix of tactics and techniques used by threat hunters, red teamers and defenders to better classify attacks and assess organizational risks.

In this paper, we have presented our research results from the association between security culture dimensions and known adversary tactics and threats. We have exhibited how a culture assessment tool aiming at different levels, dimensions and domains of cyber-security can assess the implementation status of the hybrid MITRE ATT&CK for Enterprise and ICS mitigation list. Results assist in identifying, classifying and analyzing possible security gaps or deficiencies in the infrastructure, policies, procedures and strategies of an organization by exploiting ATT&CK’s knowledge base data. Starting from a holistic cyber-security culture evaluation procedure, we manage to reach down to specialized tactics, techniques and procedures an organization lacks defense against. Thus, introducing a structured assessment approach towards identifying security deficiencies. Our research fills the gap in the current literature review where the association between security facets and potential threats was currently fragmented throughout different studies from different perspectives such as human behavior threat studies and IT security research. This paper suggests exploiting a generic cyber-security culture framework to evaluate the current security status and fulfilment of an organization and identify cyber-gaps that malicious outsiders might take advantage of.

Our ultimate goal is to assist both organizations and individuals in comprehending adversary thinking and behavioral patterns while cultivating a deeper and substantial cyber-security culture. Cyber-attacks can often be performed in a variety of ways. Consequently, blocking or detecting a single way to perform a specific technique does not necessarily mean immune against that technique. Understanding in-depth the why, how, when and who can perform a malicious act is far more beneficial than simply fighting against known classified attack techniques and procedures.

Our next steps focus on applying the suggested methodology to a number of organizations active in the EPES sector as part of the EnergyShield research project’s ongoing pilot cases [45]. Continuous effort on evolving our cyber-security culture framework while constantly keeping pace with the MITRE ATT&CK models’ updates is required. The results of our research can be utilized in an extensive set of applications and further studies, such as the efficient organization of security procedures as well as enhancing security readiness evaluation results by providing more insights into imminent threats and security risks.

## Figures and Tables

**Table 1:** MITRE ATT&CK for the Enterprise and ICS Mitigations List.

ID	Name	ATT&CK for Enterprise	ATT&CK for ICS
M0800	Authorization Enforcement		●
M0801	Access Management		●
M0802	Communication Authenticity		●
M0803	Data Loss Prevention		●
M0804	Human User Authentication		●
M0805	Mechanical Protection Layers		●
M0806	Minimize Wireless Signal Propagation		●
M0807	Network Allowlists		●
M0808	Encrypt Network Traffic		●
M0809	Operational Information Confidentiality		●
M0810	Out-of-Band Communications Channel		●
M0811	Redundancy of Service		●
M0812	Safety Instrumented Systems		●
M0813	Software Process and Device Authentication		●
M0814	Static Network Configuration		●
M0815	Watchdog Timers		●
M0816	Mitigation Limited or Not Effective		●
M1013	Application Developer Guidance	●	●
M1015	Active Directory Configuration	●	●
M1016	Vulnerability Scanning	●	●
M1017	User Training	●	●
M1018	User Account Management	●	●
M1019	Threat Intelligence Program	●	●
M1020	SSL/TLS Inspection	●	●
M1021	Restrict Web-Based Content	●	●
M1022	Restrict File and Directory Permissions	●	●
M1024	Restrict Registry Permissions	●	●
M1025	Privileged Process Integrity	●	
M1026	Privileged Account Management	●	●
M1027	Password Policies	●	●
M1028	Operating System Configuration	●	●
M1029	Remote Data Storage	●	
M1030	Network Segmentation	●	●
M1031	Network Intrusion Prevention	●	●
M1032	Multi-factor Authentication	●	●
M1033	Limit Software Installation	●	
M1034	Limit Hardware Installation	●	●
M1035	Limit Access to Resource Over Network	●	●
M1036	Account Use Policies	●	●
M1037	Filter Network Traffic	●	●
M1038	Execution Prevention	●	●
M1039	Environment Variable Permissions	●	
M1040	Behavior Prevention on Endpoint	●	
M1041	Encrypt Sensitive Information	●	●
M1042	Disable or Remove Feature or Program	●	●
M1043	Credential Access Protection	●	
M1044	Restrict Library Loading	●	●
M1045	Code Signing	●	●
M1046	Boot Integrity	●	●
M1047	Audit	●	●
M1048	Application Isolation and Sandboxing	●	●
M1049	Antivirus/Antimalware	●	●
M1050	Exploit Protection	●	●
M1051	Update Software	●	●
M1052	User Account Control	●	
M1053	Data Backup	●	●
M1054	Software Configuration	●	●
M1055	Do Not Mitigate	●	
M1056	Pre-compromise	●	

**Table 2:** Cyber-Security Culture model relation to MITRE ATT&CK for Enterprise and ICS Mitigations.

Level	Dimension	Domain	MITRE ATT&CK Mitigation
**Organizational**	**Assets**	Application Software Security	M0813
			M0815
			M1013
			M1040
			M1042
			M1045
		Data Security and Privacy	M0803
		Hardware Assets Management	M0813
			M1034
		Hardware Configuration Management	M0815
			M1024
			M1028
			M1039
			M1046
		Network Configuration Management	M0814
			M1037
		Network Infrastructure Management	M1037
		Software Assets Management	M0815
			M1033
			M1038
			M1040
			M1042
			M1044
			M1045
			M1048
			M1054
		Personnel Security	M0804
		Physical Safety and Security	M0805
			M0812
	**Continuity**	Backup Mechanisms	M1029
			M1053
		Business Continuity & Disaster Recovery	M0810
			M0811
			M1053
		Continuous Vulnerability Management	M1016
			M1051
	**Access and Trust**	Access Management	M0800
			M0801
			M1015
			M1022
			M1030
			M1035
		Account Management	M1015
			M1018
			M1032
			M1036
			M1052
		Password Robustness and Exposure	M1027
			M1043
		Privileged Account Management	M1025
			M1026
		Role Segregation	M0800
		Wireless Access Management	M0806
	**Operations**	Efficient Distinction of Development, Testing and Operational Environments	M1048
		Risk Assessment	M1019
	**Defense**	Boundary Defense	M0802
			M0807
			M0808
			M0809
			M1020
			M1031
		Cryptography	M1041
		Email and Web Browser Resilience	M1021
		Malware Defense	M1049
		Security Awareness and Training Program	M1017
	**Security Governance**	Audit Logs Management	M1047
		Penetration Tests and Red Team Exercises	M1050
**Individual**	**Behavior**	Security Behavior	M1017
	**Competency**	Security Skills Evaluation	M1017
			Μ1027
		Training Completion and Scoring	M1017

Note: ATT&CK Mitigation “***M1055—Do Not Mitigate***,” which is meant to associate with techniques that mitigation might increase risk of compromise and therefore mitigation is not recommended [18] and has been omitted from this table.

## Data Availability

Not applicable.
